# The acceptance of the clinical photographic posture assessment tool (CPPAT)

**DOI:** 10.1186/s12891-018-2272-7

**Published:** 2018-10-12

**Authors:** Carole Fortin, Paul van Schaik, Jean-François Aubin-Fournier, Josette Bettany-Saltikov, Jean-Claude Bernard, Debbie Ehrmann Feldman

**Affiliations:** 1 École de réadaptation, Faculté de médecine, Université de Montréal, C.P. 6128, succursale Centre-ville, Montréal, Québec, H3C 3J7 Canada; 2 Research center, CHU Sainte-Justine, Montreal, Quebec, Canada; 3 Department of Psychology, Teesside University, Middlesbrough, UK; 4 Institute of Health and Social Care, Teesside University, Middlesbrough, UK; 5 Centre Médico-Chirurgical de Réadaptation des Massues, Croix Rouge française, Lyon, France; 6 Institut de Recherche en santé publique de l’Université de Montréal and Centre for interdisciplinary research in rehabilitation, Montreal, Quebec, Canada

**Keywords:** Posture, Posture assessment, Musculoskeletal disorders, Technology acceptance, Innovation adoption

## Abstract

**Background:**

There is a lack of evidence-based quantitative clinical methods to adequately assess posture. Our team developed a clinical photographic posture assessment tool (CPPAT) and implemented this tool in clinical practice to standardize posture assessment. The objectives were to determine the level of acceptance of the CPPAT and to document predictors as well as facilitators of and barriers to the acceptance of this tool by clinicians doing posture re-education.

**Methods:**

This is a prospective study focussing on technology acceptance. Thirty-two clinician participants (physical therapists and sport therapists) received a 3–5 h training workshop explaining how to use the CPPAT. Over a three-month trial, they recorded time-on-task for a complete posture evaluation (photo - and photo-processing). Subsequently, participants rated their acceptance of the tool and commented on facilitators and barriers of the clinical method.

**Results:**

Twenty-three clinician participants completed the trial. They took 22 (mean) ± 10 min (SD) for photo acquisition and 36 min ± 19 min for photo-processing. Acceptance of the CPPAT was high. Perceived ease of use was an indirect predictor of intention to use, mediated by perceived usefulness. Analysis time was an indirect predictor, mediated by perceived usefulness, and a marginally significant direct predictor. Principal facilitators were objective measurements, visualization, utility, and ease of use. Barriers were time to do a complete analysis of posture, quality of human-computer interaction, non-automation of posture index calculation and photo transfer, and lack of versatility.

**Conclusion:**

The CPPAT is perceived as useful and easy to use by clinicians and may facilitate the quantitative analysis of posture. Adapting the user-interface and functionality to quantify posture may facilitate a wider adoption of the tool.

**Electronic supplementary material:**

The online version of this article (10.1186/s12891-018-2272-7) contains supplementary material, which is available to authorized users.

## Background

Physiotherapists are often consulted to assess and correct posture for persons with various musculoskeletal conditions [1, 2]. Presently, there is a lack of high-quality evidence regarding the effectiveness of physiotherapy interventions on posture [3–5]. This may be due to the lack of evidence-based quantitative clinical methods to adequately assess the outcomes of therapeutic interventions [3, 6, 7]. Currently, quantitative methods for posture assessment require elaborate 3D analysis systems such as Motion Analysis and surface topography [8, 9]. However, these systems are not easily accessible for most clinicians since they are expensive and require specialized trained technicians. Physiotherapists and physicians commonly assess posture by descriptive visual inspections that lack scientific validation [1, 10, 11]. There is a growing field of interest in using clinical tools to quantitatively assess posture. A promising technique to assess posture clinically is a method that calculates body angles and distances on photographs reflecting posture in all planes [12–15]. In recent years, different non-invasive computer-based methods as well as mobile applications (APPs) have been proposed to assess posture in a clinical setting [15–20]. Boland et al. [16] reported good intra and inter-rater agreement (ICCs ≥0.75) for seven out of 13 posture indices in ten young healthy adults using a mobile APP. Posture deviations of the head, trunk and pelvis were also measured using an iPhone APP in a large group of healthy collegiate students but the reliability and validity of such measurements are not provided. Aroeira et al. [15] reported that most of the new computer-based methods proposed in the literature to assess posture in adolescents with idiopathic scoliosis (four on 2D photogrammetry and 11 on laser or structured light, ultrasound and moiré scanner projection) focussed only on the back view and that the methodology of these studies was of low quality. These authors pointed out the importance of measuring posture of the whole body in patients with idiopathic scoliosis because the posture alterations may be extended to the whole body. Our team has developed a software program for quantitative analysis of whole body posture from digital photographs in youth with idiopathic scoliosis [18, 19]. Measures obtained using this software-based method showed excellent test-retest and inter-rater reliability for marker placement as well as good concurrent validity with spinal angles measured on radiographs and 3D trunk posture indices measured from a topography system in adolescents with idiopathic scoliosis [18, 19]. According to Aroeira et al. [15], this innovative clinical photographic posture assessment tool (CPPAT) is the only validated clinical tool offering assessment of the full body posture. The CPPAT could be used to standardize posture assessment in persons with scoliosis or other musculoskeletal pathologies.

The acceptance of rehabilitation technology by clinicians [21, 22] and patients [23] is essential for its successful uptake to both improve clinical practice as well as outcomes for patients. Previous research [22] established that drivers of the use of a low-cost portable system for postural assessment include training/skills, clinical use, quality of human-computer interaction, visualization and time-on-task; barriers to use include time-on-task, costs, quality of human-computer interaction, training/skills, clinical use, IT/equipment required and technical measurement issues.

**Fig. 1:**
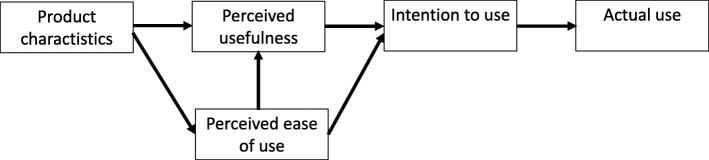
Technology acceptance model (after Davis & Venkatesh, 1996 [38])

Furthermore, it is essential to develop an understanding of how different factors influence technology acceptance. Highly influential has been Davis’s [24] technology acceptance model (TAM; see Fig. 1) and its further development [25–27]. Our study uses TAM and focuses on three core model variables: intention to use the CPPAT, perceived usefulness and perceived ease of use of the CPPAT. According to TAM, the intention to use a product (system) is the major factor influencing the extent to which potential users will employ the product (actual system use). In turn, intention to use is influenced by perceived usefulness and perceived ease of use. Perceived ease of use also indirectly influences intention to use through its direct effect on perceived usefulness. Product characteristics (system design features) directly influence both perceived usefulness and perceived ease of use and thereby indirectly influence intention to use and actual system use.

Research has also examined the relationship between task performance and perceived ease of use. Specifically, Venkatesh and Bala [25] measured ‘objective usability’ as novice-to-expert ratio of time-on-task and showed that objective usability predicts both perceived ease of use and perceived usefulness. Moreover, Chiou et al. [28] established that time-on-task predicts perceived usefulness. As task performance predicts perceived usefulness and perceived usefulness predicts intention to use, perceived usefulness may be a mediator of the effect of task performance on intention.

In our study, we address the following research questions: (1) what is the level of acceptance of this CPPAT by clinicians doing posture re-education, (2) what are the predictors of acceptance and (3) what are the drivers of and the barriers to acceptance of CCPAT for the evidence-based measurement of posture?

## Method

### Design

In a prospective mixed design study using quantitative and qualitative methods, we measured perceived ease of use, perceived usefulness and intention to use the software-based CPPAT (see Material and apparatus) for posture measurement as well as time-on-task for photo acquisition and photo-processing with the CPPAT.

### Participants

We recruited 32 clinicians (22 physical therapists and ten sports therapists) working in public (35%) or private institutions in Canada (Montreal [MTL] and Quebec city [QC]), France (Lyon) and United Kingdom (UK – London, Middlesbrough, Chesterfield). Therapists working in public centers and private clinics were invited by e-mail in order to allow the clinician participants to attend the training and the focus group discussion. Collaborators affiliated with our research teams were responsible to recruit a total of 30 therapists in the three countries. The inclusion criteria were clinicians assessing posture of persons with musculoskeletal disorders within their clinical practice and having access to a dedicated space for photo acquisitions. Eight participants did not complete the trial because they had changed their workplace and one for unknown reasons. Clinician-participants (18 women) had an average of 19.6 years (SD = 9.7) of experience in clinical practice and 12.6 years (SD = 7.7) of experience in posture assessment. In terms of computer use, ten participants had a low level, seven a moderate level, two a high level and four participants did not answer this question (see Additional file 1 for description of levels of computer use [29, 30]). The project was approved by the Institutional Ethics Committee of Sainte-Justine university hospital centre (approval reference number: 2015–691, 3905) and all clinician-participants signed a consent form.

### Materials and apparatus

#### Description of the clinical photographic posture assessment tool (CPPAT)

**Fig. 2:**
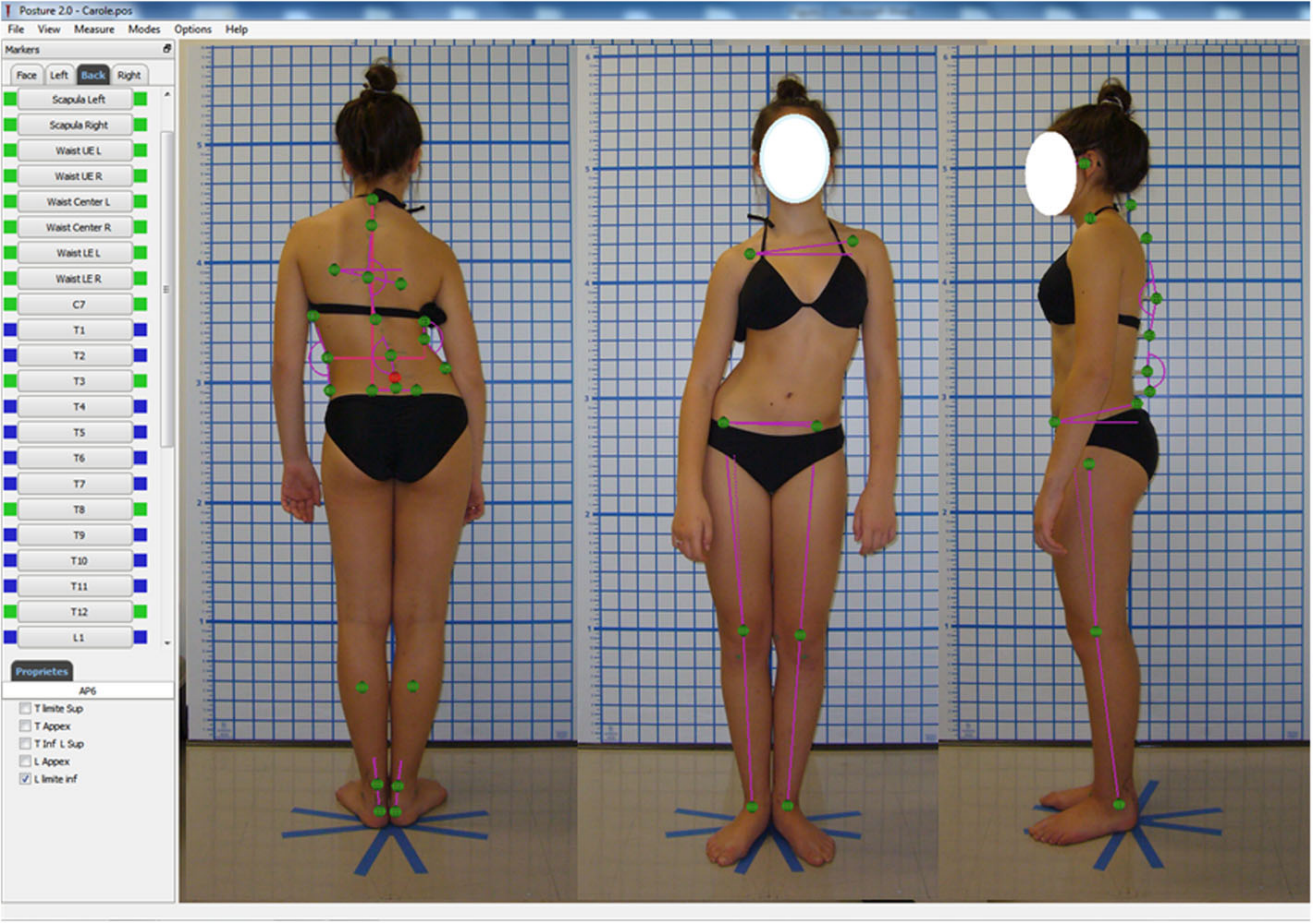
Graphical interface with photos of the clinical photographic posture assessment tool (CPPAT)

CPPAT is a software-based program with a graphical interface for the analysis of four to six photographs of a patient’s posture (front, back, left and right) acquired in standing using a standard procedure (see Fortin et al., [18, 19] for more details). We have shown excellent test-retest and inter-rater reliability for marker placement among a senior and novice physical therapists (reliability coefficients between 0.90 and 1.00 and standard error of measurement ranging from 0.5° to 3.0° and 3 to 6 mm) [18]. The software uses interactive click-on markers with the computer mouse. The user selects each specific marker from the graphical interface and places it directly on the corresponding anatomical landmark or anatomical reference points (e.g. eyes, upper end, lower end and center of the waist) on the person’s photographs (see Fig. 2). The program allows zooming in on a marker for more accuracy. Different sets of markers are available according to each view (anterior, posterior or lateral). Following the selection of the markers associated with the calculation of an angle, its value can be displayed. All measurements can be exported in Excel- or Word formats. We thus choose to study the acceptance of the CPPAT because this tool has good demonstrated reliability and validity, allows posture assessment of the whole body, was designed to be user-friendly and follows a standardized procedure.

### Procedure

The first part of the project involved the training of clinicians. The principal investigator (CF) and a research physical therapist (J-F A-F) trained clinician-participants. Participants received a tool kit including a detailed procedure for standardization of photo acquisition (following Fortin et al.’s study [18]) and of photo-processing with the software program and markers. The training consisted of a three- to five-hour workshop in each centre. The workshop was divided into three parts: 1) rationale and explanation of the software program, 2) equipment requirements (simple digital camera on a tripod) and demonstration of posture assessment with the placement of markers and photo acquisition and 3) instruction and practice using the software for photo-processing. As part of their training, clinician-participants had to use the software program to assess posture of three persons (patients or colleagues) before beginning the trial, to ensure they were familiar with the posture assessment procedure. Following the training period, participants were asked to collect the number of patients assessed with the tool and time spent for photo acquisition and for photo-processing with the software program on a data sheet for three months.

The second part consisted of a post-trial focus group discussion in small groups of up to six participants. Before beginning the discussion, participants submitted their data sheet with the number of patients analysed with the tool, together with the time for photo acquisition and photo-processing. Subsequently, van Schaik et al.’s [22] questionnaire was used to measure technology acceptance in terms of perceived ease of use, perceived usefulness and intention to use (see Additional file 2). The focus group discussion was conducted by the researchers in UK (J B-S) and in France (CF) and a physical therapist research assistant (J-F A-F) in Quebec (Canada). We used a semi-structured procedure with specific questions regarding general positive and negative aspects of the tool, its utility, utilization and patients’ feedback. Participants were also asked to comment on the advantages/disadvantages they experienced of the clinical method, drivers/barriers of system use and other possible applications of the method.

### Statistical analysis

**Fig. 3:**
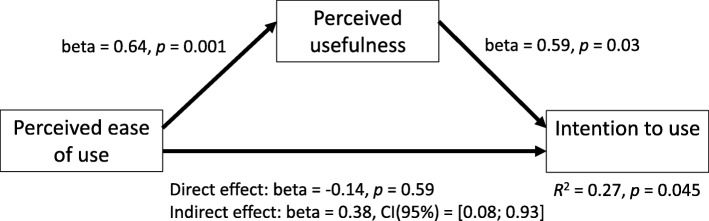
Mediation analysis (perceived ease of use)

**Fig. 4:**
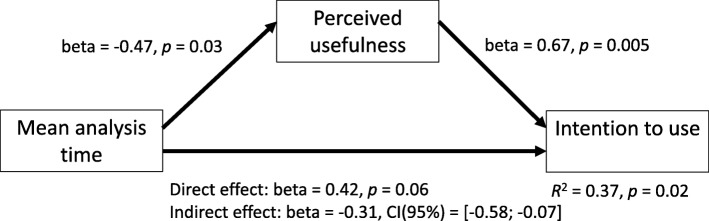
Mediation analysis (average analysis time)

Descriptive statistics (mean and *SD*), confidence intervals and *t*-tests were used to characterize the number of patients assessed with the method and time for photo acquisition and photo-processing with the CPPAT. The data were examined for normality; skew and kurtosis were not extreme (|z[skew]| < 1.8; z[kurtosis] < 1.1) and the distributions were not significantly different from the normal distribution(Komolgorov-Smirnov test: *p* > .05). t-tests were used to determine if scores obtained for each sub-scale differed from the neutral score (represented by a value of 4 on a seven-point Likert scale). We assessed reliability of each acceptance measure scale by calculating Cronbach’s alpha. Correlation analysis examined the association between the three acceptance variables. A first mediation analysis was conducted to test perceived usefulness as a potential mediator of perceived ease of use to predict intention to use the system; a second analysis tested mediation of the predictor average photo-processing time (see mediation models in Figs. 3 and 4)^1^.

A qualitative analysis was achieved using van Schaik et al.’s [22] procedure to document drivers and barriers to the acceptance of the CPPAT. The research physical therapist (J-F A-F) read all comments and initially categorised each comment into themes. He reviewed the themes again and created more general (higher-order) categories on top of the initial categories. He and the senior researcher (CF) then discussed and agreed a higher-order list of themes/categories. Both researchers independently coded all comments using the higher-order categories and recorded their codings. Finally, they compared their results, noted the number of disagreements out of the total number of codings, and discussed and resolved any disagreements.

## Results

### System use

During the course of the trial, participants assessed a mean of 7.7 patients (*SD* = 4.17, *CI*(95%) = [6.14; 9.29]). The most frequent medical diagnosis were respectively idiopathic scoliosis, back pain and hyper-kyphosis. At their first evaluation, clinician-participants took 36 min (mean, *SD* = 19, *CI*(95%) = [29; 43])^2^ for photo acquisition and 54 min (mean, *SD* = 29, *CI*(95%) = [43; 66]) for photo-processing with CPPAT. At their last evaluation, the time-on-task with the CPPAT decreased significantly, to 22 min (mean, *SD* = 10, *CI*(95%) = [18; 27], *t* (20) = 3.99, *p* = .001, *d* = 0.88) for photo acquisition and to 36 min (mean, *SD* = 19, *CI*(95%) = [29; 44], *t* (20) = 5.29, *p* < .001, *d* = 0.74) for photo-processing.

### Level of acceptance

Perceived usefulness, perceived ease of use and intention to use were measured reliably (Cronbach’s alpha = 0.89, 0.92 and 0.87, respectively).^3^ Descriptive statistics indicated that respondents believed the CPPAT was useful (mean = 5.09, *SD* = 1.05, *CI*(95%) = [4.66; 5.52]), easy to use (mean = 4.83, *SD* = 1.29, *CI*(95%) = [4.32; 5.30]) and had the intention to use the tool (mean = 4.42 (*SD* = 1.27, *CI*(95%) = [3.90; 4.98]). Perceived usefulness, perceived ease of use and intention to use were significantly higher than neutral, with a large effect size (*d* = 1.04), medium effect size (*d* = 0.64) and small effect size (*d* = 0.31), respectively.^4^

### Mediation model of technology acceptance

Correlations among the variables showed that perceived usefulness was strongly correlated with perceived ease of use (*r* = .64, *p* = .001) and intention to use (*r* = .51, *p* = .01). Therefore, as perceived usefulness and perceived ease of use increased, intention to use also increased. The correlation between perceived ease of use and intention to use was small (*r* = .24, *p* = .26). In addition, mean analysis time was strongly negatively correlated with perceived usefulness (*r* = −.47, *p* = .03) and moderately negatively correlated with perceived ease of use (*r* = −.39, *p* = .08). Therefore, as time increased, perceived usefulness and perceived ease of use decreased. The positive correlation between intention to use and analysis time was small (*r* = .11, *p* = .64).

Mediation analyses were conducted to test perceived usefulness as a mediator of the predictors (1) perceived ease of use and (2) analysis time for intention to use. In the first analysis, the mediation model was statistically significant, explaining 27% of variance in intention to use (see Fig. 3). Perceived ease of use was significant as an indirect positive predictor of intention to use, mediated by perceived usefulness. Therefore, the reason why intention to use was higher when the system was perceived to be easier to use was that it was perceived to be more useful. However, perceived ease of use was not significant as a direct predictor. According to Zhao et al.’s [31] decision tree, the pattern of results can be interpreted as indirect-only mediation: the mediator fully mediated^5^ and explained the prediction of intention to use by perceived ease of use. Apart from its function as a mediator, its significant regression coefficient on intention to use (see Fig. 3) shows that perceived usefulness was also a predictor of intention to use, independent of perceived ease of use.

The mediation model in the second analysis was also significant, explaining 37% of variance in intention to use (see Fig. 4). Analysis time was significant as an indirect negative predictor of the intention to use, mediated by perceived usefulness. Therefore, the reason why intention to use was reduced when analysis time was longer was that the system was perceived to be less useful. Analysis time was significant as a direct predictor, so mediation was partial: the prediction of intention to use by analysis time was partially mediated by perceived usefulness. According to Zhao et al.’s [31] decision tree, the pattern of results showing partial mediation can be interpreted as indicative of an incomplete theoretical framework. In other words, although part of the prediction of intention to use by analysis time was explained by the mediator perceived usefulness, in future research one or more other further mediators that were not included here may explain the significant direct prediction that was found. Apart from its function as a mediator, its significant regression coefficient on intention to use (see Fig. 4) shows that perceived usefulness also was a predictor of intention to use, independent of analysis time.

### Drivers and barriers

**Fig. 5:**
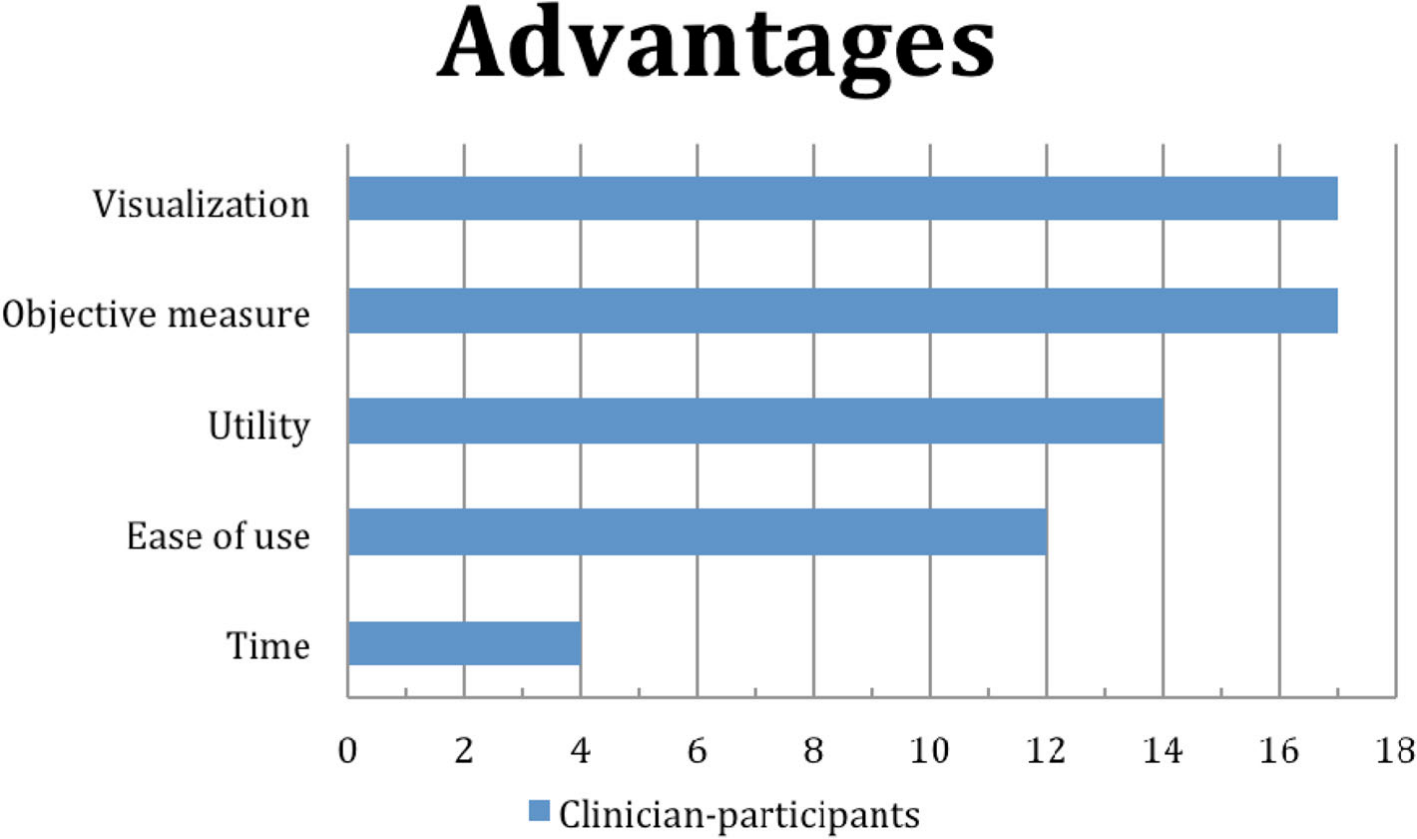
Frequencies of facilitators/advantages

**Fig. 6:**
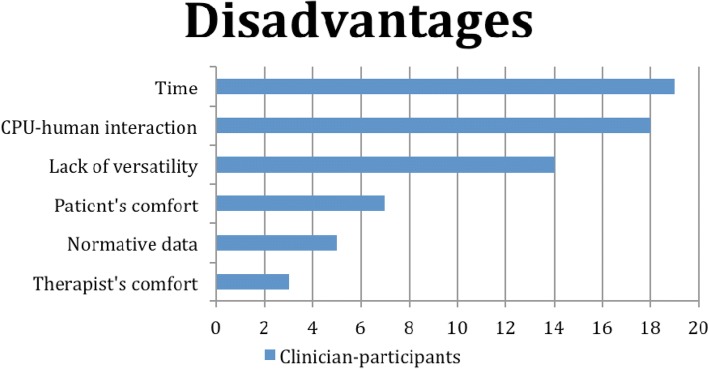
Frequencies of barriers/disadvantages

Our clinician-participants indicated four principal facilitators/advantages and four principal barriers/disadvantages. Frequencies of advantages and disadvantages are presented in Figs. 5 and 6.

#### Facilitators/advantages

Principal advantages were objective measures (17), visualization for both patients and therapists (17), utility (16), and ease of use (12). Within objective measures, accuracy of measurements and ability to document quantitative changes of posture were the most frequent answers. Regarding visualization, answers showed advantages in helping patients’ adherence to treatment, as well as guiding the therapists in seeing posture compensation. For utility, most frequent answers were useful for clinical research, as an x-ray substitute, screening tool, for patient education, treatment justification, and discussion with physicians. In terms of ease of use, the advantages were stated to be as follows: manipulation of images in the graphical interface and image processing. Four clinician-participants considered time as an advantage since they were able to achieve a complete evaluation of posture within an hour.

#### Barriers/disadvantages

The principal barriers stated were time to do a complete analysis of posture (19), the quality of human-computer interaction (18), non-automation of posture index calculation and photo transfer (18) and lack of versatility (14). Within the time category, participants included the time to take the photo, to transfer the photo into the software program, as well as processing the photo. For human-computer interaction, participants indicated that it was hard to print or copy the processed photo, the software program was only functioning on Windows systems (not on tablet, iPhone or MAC computer), it was complex to export data and the technology was complex in general for older therapists. Regarding non-automation, the most frequent answers were manual processing of the photo, a few software bugs, manual importation of photo, and manual conversion from pixels to cm for linear posture indices. In terms of versatility, being limited to four photos, all in standing and the lack of some posture indices such as head protraction in cm or not being able to add other posture indices were the most frequent comments reported. Some clinician-participants stated the absence of normative data (5) as well as the patients’ discomfort with removing clothing (7) or therapists’ comfort in terms of positioning themselves while putting the markers on anatomical landmarks of the lower extremities (3) as further disadvantages.

## Discussion

The aim of this study was to assess the acceptance of a new CPPAT among therapists who frequently assess posture as part of their clinical practice. We found strong and moderate acceptance of the CPPAT respectively in terms of usefulness and ease of use with a slightly positive intention to use the CPPAT. Our mediation analysis revealed that perceived usefulness and perceived ease of use as well as analysis time were indirect predictors of intention to use. This is in agreement with previous studies that showed the importance of these components in technology acceptance [22, 26, 27, 32].

According to Rogers [33], a new technology is more easily adopted if it is compatible with current practice, is seen as more advantageous than current practice and is easy to use (low complexity). Posture assessment was an integral part of the current practice of our participants. However, their perception of performing better in their job was divided: some saw an advantage to use the tool while others did not.

Other factors such as attitudes towards the new innovation, measurement properties of a tool, perception of self-efficacy, being able to observe its use by others and having the possibility to try it out are important for innovation adoption [33–35]. In our study, participants agreed with the good measurement properties of the tool and with the usefulness of the tool for quantifying body posture but their intention to use the tool was only slightly positive. In his model, Davis [24] pointed out that ease of use is often associated with the notion of no effort. For some participants, learning to use the tool seemed to be a greater effort than for other participants and may have led to a perception of poor self-efficacy. Indeed, several participants (*n* = 10) mentioned having a low level of computer use. Human-computer interaction and time to do a complete analysis of posture (photo acquisition and photo-processing with the CPPAT) were the most important barriers to acceptance.

In our laboratory, our research physical therapist takes on average 15 to 20 min for photo acquisition and our trained-students (same training offered to our clinician-participants) take ten to 12 min for photo-processing with the CPPAT for one complete trial. At the end of the three-month trial, 15 out of 23 (65%) and 11 out of 23 (48%) participants achieved this performance, respectively, for photo acquisition and for photo-processing with the CPPAT. With the exception of two participants, the better performance for photo acquisition was found in those participants who were used to take photos as part of their routine posture assessment of their patients.

Furthermore, some participants worked more specifically with children. It was therefore expected that it would take them more time to conduct the photo acquisition because children have more difficulty in maintaining a quiet standing posture [36]. Regarding photo-processing time, all participants conducted at least three complete evaluations of posture following their practice trial. This suggests that they had the minimal requirements to develop new skills and to achieve a good performance with the CPPAT.

Factors such as clinician-participants’ age or level of computer use may have affected task performance [34, 37]. Kaya [37] reported a negative effect of age and of low computer experience on attitudes toward computers in healthcare practitioners. Our clinician-participants had a mean of 20 years of experience in clinical practice and eight out of the ten participants who had taken a longer time for photo-processing with the CPPAT had a low level of computer use, which may explain their difficulty in performing better as well as their low level of interest to use the tool. This may also explain why for half of our participants the graphical interface of the software program was seen as user-friendly while not for the others.

Other barriers for the CPPAT acceptance were the lack of automation of posture index calculation and photo transfer, and the lack of versatility of the tool in terms of positions of posture acquisition and computer operating system. Further development of the tool focusing on automation of photo transfer and posture index analysis would contribute to a substantially decreased time for photo-processing and may thus promote an increased adoption of the tool. Some participants mentioned that they could not take photos in other positions apart from standing or could not add new posture indices. In the present study, they were asked to take photos in a standing position but we showed in a previous study that it is also possible to take and analyse photos in a sitting position [17]. Moreover, new posture indices would be easy to implement in a new version of the tool.

### Limitations

The main limitation of our study was the small number of participants that completed the trial. However, this sample size was large enough to demonstrate a high level of perceived ease of use and perceived usefulness and demonstrate statistical mediation, and identify the main barriers for the CPPAT acceptance. Moreover, the sample size in each country was too small to formally compare the results between countries. The two participants in France were familiar with sophisticated systems to measure static and dynamic posture. Hence, they both found the tool easy to use and user-friendly. Participants from UK and Quebec (Canada) were more heterogeneous and tend to show similar results in the acceptance of the tool.

We also acknowledge that some participants did not have easy access to a dedicated space for photo acquisitions even though this was an inclusion criterion. Moreover, a non-facilitating environment including the absence of local champions is an important barrier and may affect innovation adoption [33, 35]. Although we had identified champions in several centres before the study began, for several reasons, these persons could not act as champion in their respective centre. A local champion might have helped in resolving problems such as the accessibility to a dedicated space for photo acquisitions or minor bugs with the software program. Attitudes towards the new technology and self-efficacy are also important factors for innovation adoption [32, 34]. In this study, we did not directly measure these factors and we did not use a validated questionnaire to measure the level of computer use. This will need to be done in a future study. Selection bias may have occurred since some clinician-participants knew the researchers and the physician leading this project. However, the answers of the clinician-participants seem to objectively reflect their ‘true’ acceptance of the tool*.*

### Clinical implications

This study highlights the usefulness of the CPPAT for quantifying posture in a clinical setting. The majority of our participants found this tool useful to document quantitative changes of posture, for a complete or partial evaluation of posture, as a screening tool, for patient education as well as for treatment justification and for discussion with physicians. According to our participants, photos allow visualization of posture, which is perceived as a good means to help patients’ adherence to treatment and guiding therapists in seeing posture compensation. Participants used the tool among persons presenting with spinal deformities such as scoliosis, hyper-kyphosis or hyper-lordosis, with back pain and lower-limb impairments. Taking photos facilitated the measurement of several body angles at a time and is more accurate and rapid than measuring direct body angles on a person, especially in those with back pain [13]. Few participants mentioned the need to have the software program functioning on Windows systems as well as on tablet, iPhone or MAC computer. Other mobile APPs have been developed to measure posture and showed promising results, but posture indices measurement errors of these APPs and their validity still need to be documented [16, 20]. Some participants also indicated that less than 30 min should be taken for a complete assessment of posture. Being able to integrate automation of photo transfer and of posture index calculation into the CPPAT should allow clinicians to have a more efficient tool and may promote adherence to this tool. To be more cost- and time-effective, clinicians may also select a set of relevant posture indices according to a patient’s condition to document change in posture over time. However, clinicians should interpret changes in posture over time with caution since reliability and validity of posture indices measurements of the CPPAT have been reported only in adolescents with idiopathic scoliosis and sensitivity to change of these posture indices measurements is not yet determined.

## Conclusion

Our results indicate that the CPPAT is perceived to be useful and easy to use by clinicians. The CPPAT tool contributes to clinical practice by facilitating the quantitative analysis of posture and by enhancing the education of patients presenting with different musculoskeletal impairments. The principal barriers for the acceptance of CPPAT were the time to conduct a complete postural analysis and difficulties in interacting with the system. Adapting the software-human interface and automation for posture index calculation may facilitate the wider adoption of the tool.

## Additional files

Additional file 1: Level of computer use: description of each level of computer use. (DOC 27 kb)

Additional file 2: Questionnaire Technology acceptance: items for perceived ease of use, perceived usefulness and intention to use. (DOC 33 kb)

## Abbreviations

CPPAT: Clinical Photographic Posture Assessment Tool
TAM: technology acceptance model

## Footnotes

1 In a mediation model, the followings effects are analysed: (1) the effect of the mediator (e.g., perceived usefulness) on the outcome (e.g., intention to use), with the predictor (e.g., perceived ease of use) held constant, (2) the direct effect of the predictor on the outcome with the mediator held constant and (3) the indirect effect of the predictor on the outcome through the mediator.

2 Bias-corrected accelerated confidence intervals are presented, with *N* = 1000 bootstrap samples.

3 For ease of interpretation of the results, average scores on each scale were calculated, reverse-scored and then used in subsequent analysis.

4 Cohen’s (1988) conventions for effect size were used for *d* (small: ±0.2; medium: ±0.5; large: ±0.8) and *r* (small: ±0.1; medium: ±0.3; large: ±0.5).

5 Full mediation occurs when the regression coefficient of the predictor (e.g. perceived ease of use) on the outcome variable (e.g. intention to use) becomes non-significant when the mediator (e.g., perceived usefulness) is introduced.
