# Supplementary material for: The acceptance of the clinical photographic posture assessment tool (CPPAT)
Source: BMC Musculoskelet Disord. 2018 Oct 12;19:366. doi: 10.1186/s12891-018-2272-7 (PMC6182862; doi:10.1186/s12891-018-2272-7)
Supplement: Supplementary file 1 — Level of computer use: description of each level of computer use. (DOC 27 kb) [file 12891_2018_2272_MOESM1_ESM.doc]

# Additional file 1

**Level of computer use***

# Level 1 (low): Using basic equipement controlled by computer :

# Input basic commands, without the need to understand how the software works.

# Perform searches on general databases (ex : Google, Bing, Yahoo).

# Send/receive an email (text).

**Level 2 (moderate):** perform tasks involving many functions :

- - Perform Word text page layout, create Power Point presentation.
  - Perform searches on specialized databases (ex : PubMed, CINAHL, PEDro).
  - Converting files from one format to another.
  - Installation of a software using a setup wizard.
  - Recognize and save files in various formats (.bmp, .jpg, .pdf, .xls, etc...).
  - Create folders to save and organize files.

**Level 3 (high):** Perform tasks involving many operations:

- - Install and configure a software using custom settings.
  - Similar tasks as at Level 2, but trial and error, and troubleshooting is necessary to obtain wanted results.
  - Basic knowledge in programming and coding.

***** Questionnaire designed with criteria from the Northstar basic computer skills certificate ^29^ and from Illinois Valley Community College^30^.
