# Supplementary material for: The acceptance of the clinical photographic posture assessment tool (CPPAT)
Source: BMC Musculoskelet Disord. 2018 Oct 12;19:366. doi: 10.1186/s12891-018-2272-7 (PMC6182862; doi:10.1186/s12891-018-2272-7)
Supplement: Supplementary file 2 — Questionnaire Technology acceptance: items for perceived ease of use, perceived usefulness and intention to use. (DOC 33 kb) [file 12891_2018_2272_MOESM2_ESM.doc]

# Additional file 2 – Questionnaire Technology acceptance – Items

## Perceived ease of use

1. Learning to use the system would be easy for me.

2. I would find it easy to get the system to do what I want it to do.

3. Interacting with the system would be clear and understandable to me.

4. I would find the system flexible to interact with.

5. It would be easy for me to become skilful at using the system.

6. I would find the system easy to use.

*Response format for all perceived-ease-of-use items*:

| Likely |  |  |  |  |  |  | Unlikely |
| --- | --- | --- | --- | --- | --- | --- | --- |
| extremely | quite | slightly | neither |  | slightly | quite | extremely |

## Perceived usefulness

1. Using the system in my job would enable me to assess back shape/posture more accurately.

2. Using the system in my job would enable me to assess back shape/posture more objectively.

3. Using the system would improve my job performance.

4. Using the system would enhance my effectiveness on the job.

5. Using the system in my job would enable me to provide better evidence for my postural assessment

6. I would find the system useful in my job.

*Response format for all perceived-usefulness items*:

| Likely |  |  |  |  |  | Unlikely |
| --- | --- | --- | --- | --- | --- | --- |
| extremely | quite | slightly | neither | slightly | quite | extremely |

## Intention to use

1. I intend to use the system for my postural assessments in the spinal unit.

Definitely do not 1 2 3 4 5 6 7 Definitely do

2. I plan to use the system for my postural assessments in the spinal unit.

Definitely do not 1 2 3 4 5 6 7 Definitely do

3. I would like to use the system for my postural assessments in the spinal unit.

Definitely yes 1 2 3 4 5 6 7 Definitely no

4. I want to use the system for my postural assessments in the spinal unit.

Strongly agree 1 2 3 4 5 6 7 Strongly disagree

5. I expect to use the system for my postural assessments in the spinal unit.

Unlikely 1 2 3 4 5 6 7 Likely

6. How likely is it that you would use the system for your postural assessments in the spinal unit?

Unlikely 1 2 3 4 5 6 7 Likely
